# Supplementary material for: Mapping the evolution of fertility support policies in China: A content and instrumental analysis
Source: PLoS One. 2025 Oct 9;20(10):e0332137. doi: 10.1371/journal.pone.0332137 (PMC12510515; doi:10.1371/journal.pone.0332137)
Supplement: S1 Appendix — (ZIP) [file pone.0332137.s001.zip › S1 Appendix. 226 original policy documents/134-邮电女职工劳动保护规定实施细则(FBM-CLI-4-27211).doc]

邮电女职工劳动保护规定实施细则

**发布部门：** [信息产业部(含邮电部)(已撤销)](https://www.pkulaw.com/law/chl?Aggs.IssueDepartment=60217) [机构沿革](javascript:void(0))

**发布日期：**1991.06.03

**实施日期：**1991.07.01

**时效性：** [现行有效](https://www.pkulaw.com/law/chl?Aggs.TimelinessDic=01)

**效力级别：** [部门规章](https://www.pkulaw.com/law/chl?Aggs.EffectivenessDic=XE0301)

**法规类别：** [劳动安全与劳动保护](https://www.pkulaw.com/law/chl?Aggs.Category=08706)

邮电女职工劳动保护规定实施细则
（１９９１年６月３日　邮电部）

　　**第一条**　为维护女职工的合法权益，减少和解决女职工在通信生产和工作中因生理特点造成的特殊困难，保护女职工的安全和健康，促进邮电通信事业的发展，根据国务院《[女职工劳动保护规定](https://www.pkulaw.com/chl/133408887a349cdabdfb.html)》和国家有关女工保护法令、规定，结合邮电实际，制定本实施细则。

　　**第二条**　本细则适用于全国邮电企业、事业单位。集体所有制单位参照执行。

　　**第三条**　凡适合妇女从事生产劳动的单位，不得拒绝招收女职工。

　　**第四条**　各单位必须严格执行上级有关劳动保护的法规和政策，不得在女职工怀孕期、产期、哺乳期降低其基本工资、停薪或解除劳动合同，不得以女职工上述生理特点为由，将其转为待聘、编余人员或辞退。

　　**第五条**　在优化劳动组合中，对那些劳动态度好、业务熟练、遵章守纪，能够完成正常生产任务的女职工，各单位应予组合。

　　**第六条**　禁止安排女职工从事电信线务高空作业、邮件押运工作，以及连续负重超过二十公斤，间断负重超过二十五公斤、国家规定的第四级体力劳动强度的劳动和对女性生理机构有特殊危害的其它女职工禁忌从事的劳动。

　　**第七条**　女职工在月经期间，所在单位不得安排从事高温、低温、低湿、冷水和国家规定的第三级体力劳动强度的劳动、经常接触超过国家规定的最高容许浓度的铅烟或烟尘及用纯苯作溶剂或原料的生产作业。

　　女职工因月经严重异常不能胜任原繁重体力劳动的，应根据医务部门证明，暂时调换轻便工作。女投递员和女卡车司机在月经期间反应较大的，可调做一至二天内勤工作。

　　**第八条**　对怀孕的女职工经常从事弯腰、攀高、下蹲、抬高和有毒有害、剧烈振动等作业，容易引起流产、早产的应暂时调换其它工作。

　　女职工怀孕期间，所在单位不得在正常劳动日以外延长劳动时间，对不能胜任原劳动的，应根据医务部门证明，予以减轻劳动量或安排其它劳动。

　　怀孕七个月以上的女职工，一般不得安排从事夜班劳动。在劳动时间内应当安排一定的休息时间并减少一定劳动量。

　　怀孕女职工在当班时间内进行产前检查，所需时间按出勤计算。

　　**第九条**　女职工怀孕流产时，应根据医务部门的证明，怀孕在两个月以内给予十五天产假；怀孕在两个月至三个月期间给予二十天产假；怀孕在三个月以上给予三十天产假；怀孕在四个月以上给予四十二天产假；怀孕在七个月以上给予九十天产假。产假期间，工资照发。

　　**第十条**　女职工怀孕，在本单位的医疗机构或者指定的医疗机构检查和分娩时，其检查费、接生费、手续费、住院费、药费由所在单位负责，费用由原医疗经费渠道开支。

　　**第十一条**　女职工产假为九十天，其中产前假十五天，难产的增加产假十五天，多胞胎生育的，每多生育一个婴儿增加产假十五天。教师符合计划生育规定的产假（含独生子女优待产假）正值寒暑假期间时，可顺延其休假时间，工资照发。女职工在休产假时，男职工可以二至四天的护理假，护理假期间待遇按公假处理。

　　符合晚育条件的女职工，除国家规定的产假外，所增产假可按地方政府计划生育有关规定执行。

　　**第十二条**　有不满一周岁婴儿的女职工，其所在单位应当在每班劳动时间内给予两次哺乳（含人工喂养）时间，每次三十分钟。多胞胎生育的，每多哺乳一个婴儿，每次哺乳时间增加三十分钟。女职工每班劳动时间内的两次哺乳时间，可以合并使用。哺乳时间和在本单位内哺乳的途中往返时间，算作劳动时间。

　　**第十三条**　女职工在哺乳期内，所在单位不得安排其从事有毒、有害、高温、低温以及国家规定的第三级体力劳动强度的劳动，不得延长劳动时间，一般不得安排其从事夜班劳动或安排加班加点。

　　**第十四条**　生产人员各项假期缺勤的备员，女职工一般按百分之八的比例计算，但县以上局的女投递员、女话务员备员，可按百分之十的比例计算。

　　**第十五条**　女职工集中的单位应当根据国家有关规定，逐步建立和完善女职工卫生室、孕妇休息室、哺乳室、托儿所、幼儿园等设施；女职工不足一百人的单位，应设置简易温水箱及冲洗器，也可发给个人自用冲洗器具，对保护女职工所需的经费应予保证。农村、偏远地区，没有条件设哺乳室、托儿所，女职工子女在五周岁以下，送局外入托，单位可酌情补助或报销一定的托儿费。

　　女职工劳动卫生费为每月四至六元。

　　**第十六条**　各单位应有计划地安排女职工每两年免费进行一次妇科病普查，四十五岁以上的女职工每年进行一次，对患病者及时给予治疗。对县或县级以上医疗机构确诊患更年期综合症的女职工，应给予照顾。

　　**第十七条**　各单位应根据女工人数多少配备专职或兼职人员负责女职工劳动保护工作，并根据女职工的生理特点和职业特点，对女职工进行安全卫生知识方面的教育。

　　**第十八条**　女职工劳动保护的权益受到侵害时，有权向所在单位主管部门或上级劳动部门提出申诉；上述部门应当自收到申诉书之日起三十日内作出处理决定；女职工对处理决定不服的，可以在收到处理决定书之日起十五日内向人民法院提起诉讼。

　　**第十九条**　对违反规定侵害女职工劳动保护权益的单位负责人及其直接责任人员，其所在单位的上级主管部门，应当根据情节的轻重，给予批评教育或行政处分，并责令该单位给予被侵害女职工合理的经济补偿；构成犯罪的，由司法机关依法追究刑事责任。

　　**第二十条**　各级劳动保护部门负责对本细则的执行情况进行检查。各级工会组织有权对本细则的执行进行监督。

　　**第二十一条**　女职工怀孕、生育等方面，涉及到计划生育政策的，按国家计划生育的有关规定处理。

　　**第二十二条**　本实施细则由邮电部劳动工资司负责解释。

　　**第二十三条**　本实施细则自一九九一年七月一日起实行。

© 北大法宝：（[www.pkulaw.com](https://www.pkulaw.com/)) 专业提供法律信息、法学知识和法律软件领域各类解决方案。北大法宝为您提供丰富的参考资料，正式引用法规条文时请与标准文本核对。
欢迎查看所有[产品和服务](https://www.pkulaw.com/helps/2.html)。法宝快讯：[如何快速找到您需要的检索结果？](https://www.pkulaw.com/helps/70.html)[法宝](https://www.pkulaw.com/helps/69.html)V6有何新特色？

扫描二维码阅读原文

**原文链接：**<https://www.pkulaw.com/chl/83955367a88bfe6dbdfb.html>
